# Case Report: Extra skeletal Ewing’s sarcoma of mediastinum: clinical experience and literature review

**DOI:** 10.3389/fonc.2025.1601648

**Published:** 2025-11-05

**Authors:** Xianwen Hu, Wei Zhao, Ronghua Yu, Dongfeng Pan

**Affiliations:** ^1^ Department of Nuclear Medicine, Affiliated Hospital of Zunyi Medical University, Zunyi, China; ^2^ Department of Pathology, Affiliated Hospital of Zunyi Medical University, Zunyi, China

**Keywords:** Ewing’s sarcoma, mediastinum, computed tomography, magnetic resonance imaging, imaging study

## Abstract

Extraskeletal Ewing sarcoma (EES) is a malignant tumor that arises in soft tissues outside the skeleton. It commonly involves the paravertebral regions, the lower extremities, and the chest wall, with mediastinal involvement being less frequent. Here, we report an 11-year-old male with pathologically confirmed EES occurring in the mediastinum. Chest computed tomography (CT) showed a large soft tissue density mass on his left posterior mediastinum, containing internal low-density cystic necrotic areas. The solid component was isointense to muscle on T1-weighted imaging (T1WI) and mildly hyperintense on T2-weighted imaging (T2WI), whereas the cystic components were hyperintense on T2WI and variably hyperintense on T1WI. On contrast-enhanced CT and T1WI, the mass demonstrated heterogeneous, progressive enhancement, suggesting the possibility of malignant tumor. A needle biopsy confirmed the diagnosis of EES. After diagnosis, the patient received systemic chemotherapy followed by surgical resection of the tumor. We also conducted a systematic review of the published literature on mediastinal EES, summarizing its clinical and imaging features, with the aim of increasing understanding of this rare disease.

## Introduction

Ewing’s sarcoma (EWS) is a highly malignant small round cell tumor of neuroectodermal origin with multipotent differentiation potential and belongs to the primitive neuroectodermal tumor (PNET) family (1). Extraskeletal Ewing sarcoma (EES) arises in soft tissues outside bone and accounts for about 15% of ES; it most commonly occurs in the paravertebral regions, lower extremities, and chest wall, while mediastinal involvement is rare (2). It is more common in children and adolescents, with 70% of patients having an onset age below 20 years old, and males more frequently affected than females (3). In the early stages, clinical symptoms are nonspecific and difficult to detect; as the tumor enlarges and compresses adjacent structures, symptoms gradually appear (3). EWS grows rapidly, and early diagnosis is seldom achieved; by the time of diagnosis, tumors are often large, with maximum diameters commonly reaching about 10 cm (4). Imaging findings vary greatly with tumor site. Because primary mediastinal ES is rare, only a limited number of radiologic studies have described its imaging features. Here, we report an 11-year-old boy with pathologically confirmed mediastinal EWS. In addition, we review the published literature on mediastinal EWS, summarize its imaging and clinical features in this unusual location, and discuss its imaging differential diagnosis in detail, with the aim of increasing understanding of this rare disease.

## Case presentation

**Figure 1:**
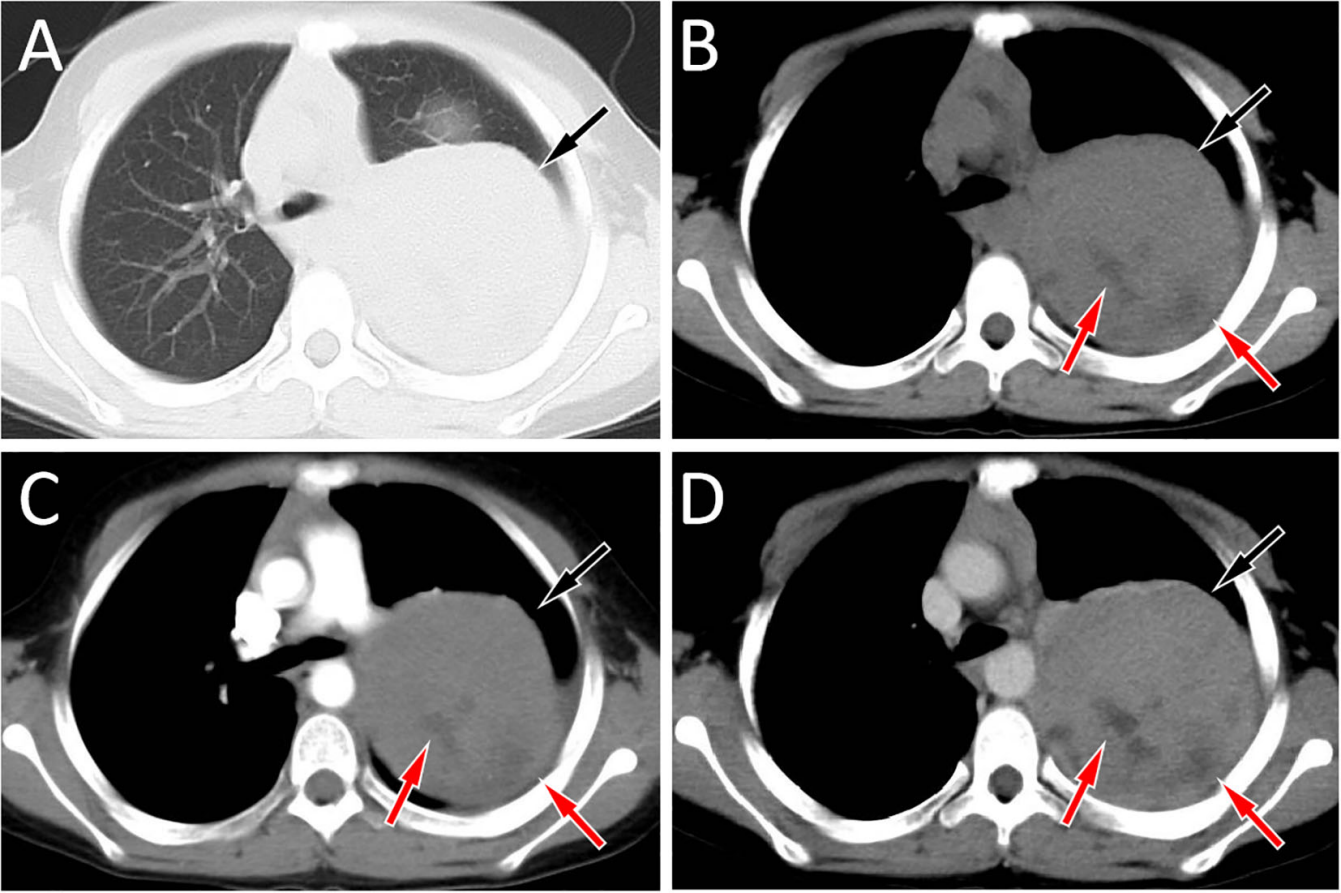
Chest computed tomography (CT) lung window **(A)** and mediastinal window **(B)** revealed a soft tissue density mass (black arrows) about 8.5 cm×7.2 cm×9.6 cm in size with low density cystic necrosis area (red arrow) can be seen on the left posterior mediastinum, and the mean CT value of the mass was 54 HU; In the arterial phase **(C)** of contrast-enhanced CT, the lesion showed mild uneven enhancement (black arrow), with a CT value of 65 HU; In the vein phase **(D)**, the enhancement degree of the mass was further enhanced (black arrow), and the CT value was 81 HU. There was no obvious enhancement in the necrotic area of cystic lesion in either arterial or venous stage (red arrows).

**Figure 2:**
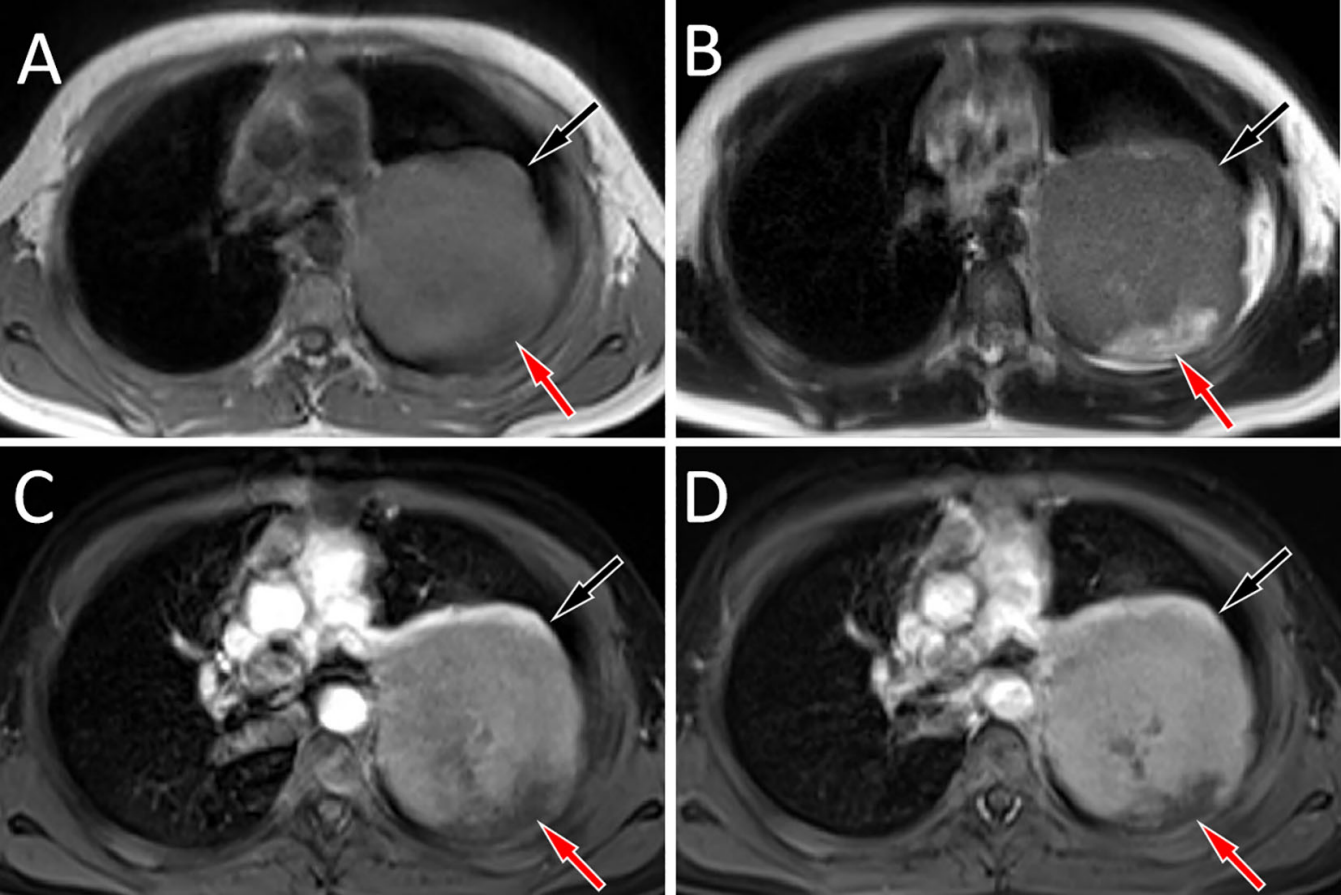
Chest MRI revealed that the mass showed equal muscle signal on T1WI [**(A)**, black arrow], slightly higher signal on T2WI [**(B)**, black arrow], and the cystic necrosis area inside showed long T1 and long T2 signals (red arrows). Contrast-enhanced T1WI reveals that the solid components of the mass show significant progressive enhancement (black arrows) in the arterial **(C)** and venous phases **(D)**, while there is no significant enhancement in the cystic necrotic area (red arrows).

**Figure 3:**
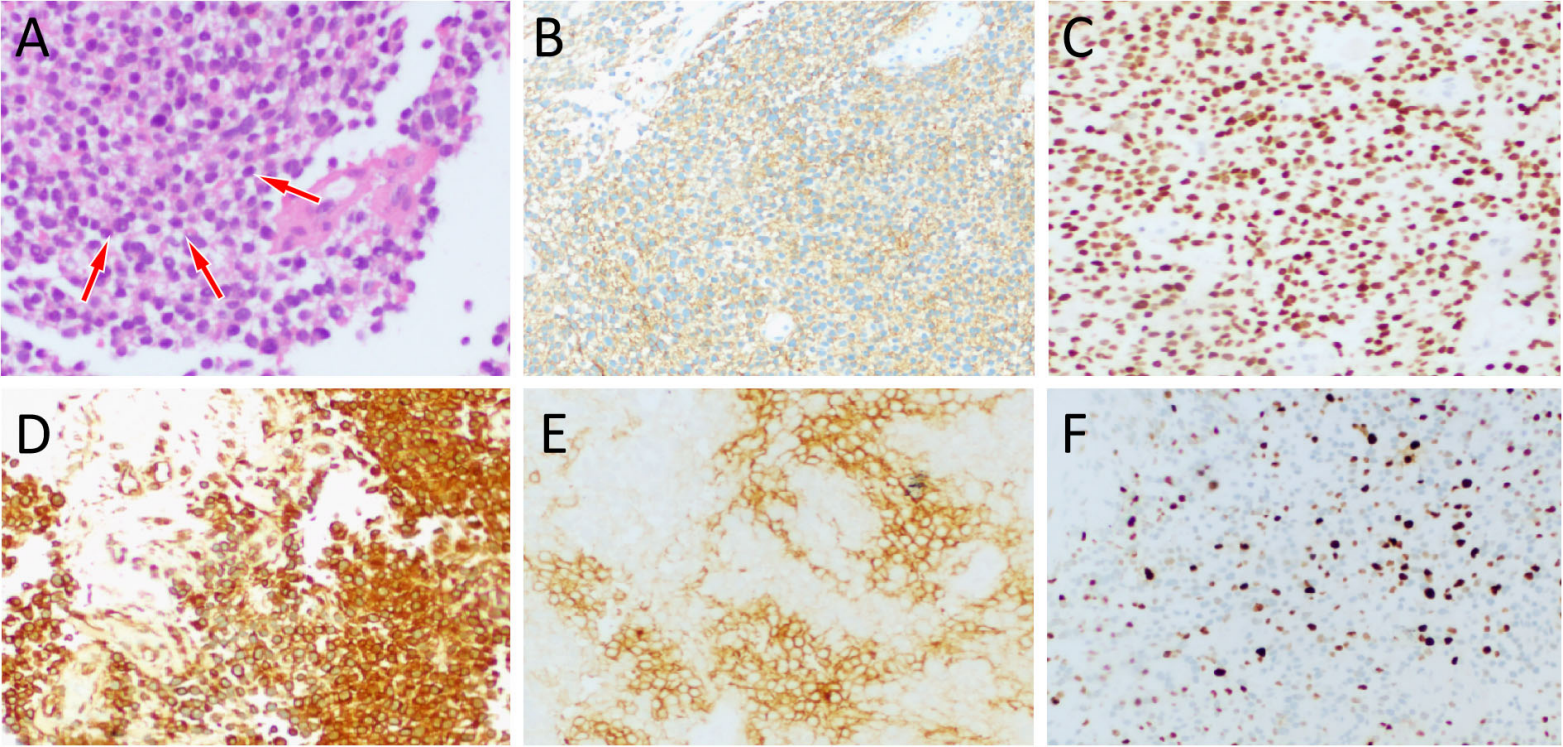
**(A)** Hematoxylin-eosin staining (magnification, ×100) showed that the tumor tissue is composed of small circular tumor cells (arrows) of similar size and shape arranged closely. Immunohistochemical results revealed that the tumor cells positively expressed CD99 **(B)**, NKX2.2 **(C)**, vimentin **(D)**, CD117 **(E)** and Ki-67 [**(F)**, about 20%].

**Figure 4:**
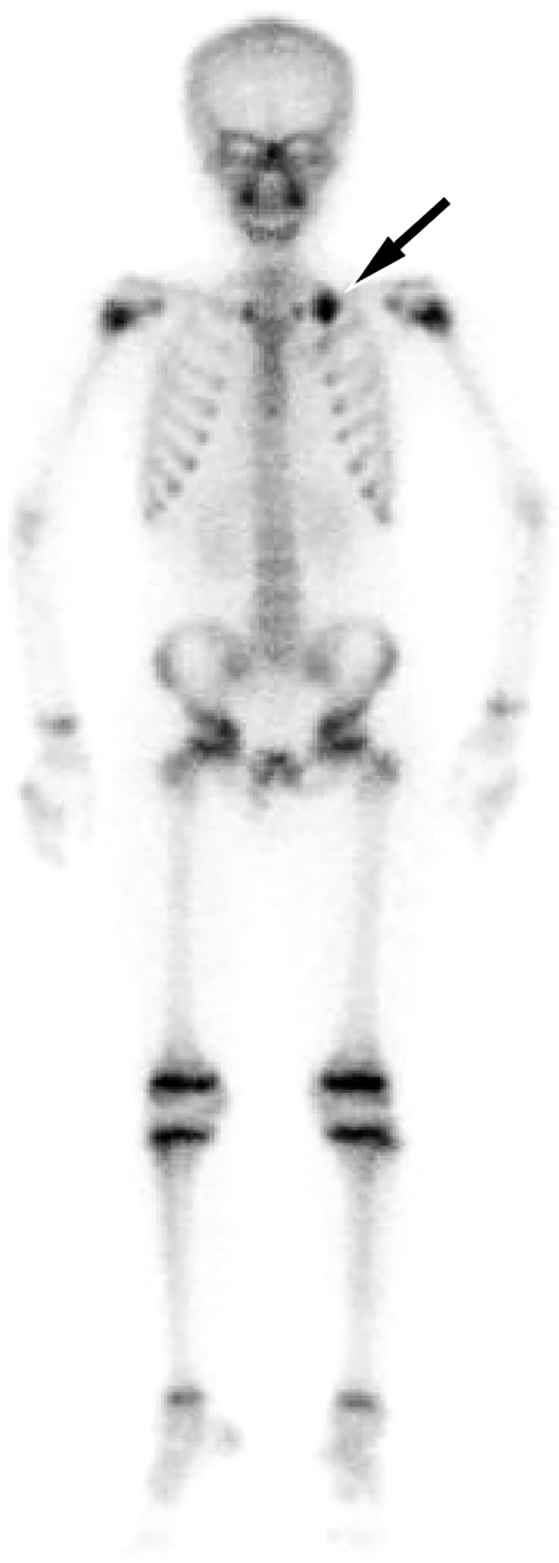
Technetium-99 labeled methylene diphosphate (^99m^Tc-MDP) whole body bone imaging revealed a concentration of radioactive tracer uptake at the left first rib of the patient (arrow), suggesting tumor invasion of it.

An 11-year-old male patient presented to our hospital on March 20, 2022 due to intermittent pain in his left shoulder for more than 6 months. Physical examination revealed left shoulder tenderness upon palpation, diminished breath sounds in the left lung field, and a small amount of wet rales could be heard. The serum tumor marker test revealed an increase in carbohydrate antigen 125 (Ca125) at 64 U/mL, while the results of other tumor marker tests were negative. The patient’s family did not report any psychological history of the patient. Both the patient and his parents denied having a history of tumor, hepatitis, tuberculosis and other serious medical history. On the day of presentation, the patient underwent a chest X-ray examination, which revealed a large lamellar dense shadow in the left upper lung field, with the mediastinum displaced to the right by compression. To further delineate the anatomic location and evaluate the nature of the lesion, the patient underwent chest computed tomography (CT) and magnetic resonance imaging (MRI) in the following days. A chest CT examination revealed a large soft tissue density mass located on the left side of the mediastinum, measuring about 85 mm×72 mm×96 mm, with low-density cystic necrotic areas visible within the mass. Contrast-enhanced CT demonstrated heterogeneous and progressive enhancement of the mass (as shown in **Figure 1**). Chest MRI examination showed that the majority of the mass presented equal T1 and slightly long T2 signals with muscle tissue, and the cystic necrosis area showed slightly long T1 and long T2 signals (**Figure 2**). According to these imaging findings, the possibility of malignant tumor was suggested. He subsequently underwent a CT-guided targeted puncture of the mass. The puncture needle was introduced into the mass via the left anterior chest wall. Histopathological examination (**Figure 3**) revealed that the tumor was composed of small round cells of similar size and shape under the microscope. The nucleus was round, with fine chromatin and many mitotic figures. The cytoplasm was sparse, accompanied by hemorrhage and necrosis. Immunohistochemical results revealed that the tumor cells expressed CD99, NKX 2.2, CD117 and vimentin positively, but negatively expressed CK, S-100, CD56. The proliferation marker Ki-67 was found to be approximately 20%. Fluorescence *in situ* hybridization revealed that the EWSR1-FLI1 gene fusion was positive. Based on these histopathological features, the patient was diagnosed with Classic Ewing’s sarcoma. In order to determine the treatment plan, the patient underwent a technetium-99-labeled methylene diphosphate (^99m^Tc-MDP) whole-body bone imaging examination (By injecting the imaging agent ^99m^Tc-MDP intravenously and utilizing single photon emission computed tomography equipment to capture the uptake and distribution of the agent within the bones, a comprehensive whole-body bone image is generated) on March 28th, which showed that the tumor had invaded the left first rib (**Figure 4**). These imaging examination results indicate that surgical resection of the tumor is not possible at this stage. The clinical doctor performed a chemotherapy regimen alternating between the VDC regimen of vincristine, doxorubicin, and cyclophosphamide and the IE regimen of ifosfamide and etoposide on the patient. Following the completion of five cycles of chemotherapy, a chest CT scan was performed on July 4, 2022. The results revealed a slight decrease in tumor size, measuring about 78 mm×65 mm×78 mm, indicating partial remission in response to treatment. After mutual consultation among clinical doctors and communication with the patient’s family, the patient underwent surgery to remove the tumor under general anesthesia on July 25. As the original chemotherapy regimen had a certain therapeutic effect on the patient, the patient continued to follow the original chemotherapy regimen after the operation until November 5, 2022. During the chemotherapy process, a grade 3 toxic side effect occurred, including a decrease in white blood cells and liver dysfunction. In response to the above situation, chemotherapy is suspended and granulocyte colony-stimulating factor (G-CSF) is given to stimulate bone marrow hematopoiesis to treat neutropenia, while compound glycyrrhetinic acid is used for liver protection and enzyme reduction to treat liver dysfunction. The patient was discharged from the hospital in December 2022 and then transferred to the affiliated hospital of Peking University Medical College for treatment with oral amrubicin hydrochloride until August 2023. After the treatment, two chest CT examinations were conducted. The most recent one was conducted on September 12th, 2023, and no obvious abnormalities were found.

After being discharged in September 2023, the patient did not undergo any further treatment until October 5, 2024, when he developed a fever and returned to Beijing Medical University Affiliated Hospital. Subsequent examinations indicated tumor recurrence. The patient underwent eight cycles of eribulin mesylate chemotherapy, followed by three cycles of mitoxantrone liposome chemotherapy, concurrently with oral lenvatinib mesylate capsules until June 2025. On July 4, 2025, the patient presented with recurrent fever and sought medical care at our hospital. Chest CT revealed multiple nodules on the left pleura and enlarged lymph nodes in the left axillary region. These lesions are all new compared to previous CT scans, so it is highly suspected to be tumor metastasis. To further assess the nature of the lesion and the patient’s whole body condition, the clinician recommended that the patient undergo a whole body positron emission tomography (PET)/CT examination. However, the patient’s family, due to financial difficulties, did not proceed with it. Then the hematologist in our hospital advised the patient’s family to allow the patient to continue chemotherapy, but the family refused. After the patient’s condition improved with symptomatic treatments such as anti-infection therapy, he was discharged from the hospital. In our most recent telephone follow-up, the patient’s family informed us that the patient had passed away in mid-August. A detailed summary of the patient’s timeline is shown in **Table 1**.

**Table 1:** The summary of the patient’s timeine.

Timeline	Affair
20/3/2022	The patient presented with intermittent pain in his left shoulder more than 6 months; Chest X-ray examination revealed a large lamellar dense shadow in his left upper lung field
21/3/2022-22/3/2022	Chest CT and MRI examination revealed a large mass on the left side of the mediastinum, suspected to be malignant
24/3/2022-27/3/2022	Puncture biopsy and pathological histological examination; Diagnosed with Ewing’s sarcoma
28/3/2022	^99m^Tc-MDP whole body bone scan revealed that the tumor invaded the left first rib
29/3/2022-4/7/2022	The patient received chemotherapy; After completing 5 chemotherapy courses, a chest CT scan revealed that the tumor volume had slightly decreased
25/7/2022	Surgical removal of the tumor for treatment
1/8/2022-5/11/2022	The patient received chemotherapy again; A grade 3 toxic side effect occurred, but it was relieved after symptomatic treatment.
3/12/2022-15/8/2023	The patient was transferred to the affiliated hospital of Peking University for treatment multiple times
12/9/2023-4/10/2024	The patient discharged, not receiving any treatment
5/10/2024	He presented with fever and sought medical attention at Beijing Medical University Affiliated Hospital; Tumor recurrence
6/10/2024-10/6/2025	Received chemotherapy
4/7/2025	The patient returned to our hospital for treatment due to recurrent fever; Chest CT examination revealed multiple metastases in the pleura and lymph nodes, but the patient’s family refused chemotherapy.
Mid-August 2025	Patient death

## Literature review

A comprehensive search of PubMed, Web of Science, and Embase identified primary mediastinal extraskeletal Ewing sarcoma (EES) case reports and case series published through November 1, 2024. The language limit was English. The search used keywords including Ewing sarcoma, primitive neuroectodermal tumors, mediastinum, and mediastinal. For each eligible case, data were extracted on the first author, year of publication, country of origin, patient sex and age, presenting clinical symptoms, imaging findings (CT and MRI), treatment modalities, and follow-up outcomes, as summarized in **Table 2**. After a systematic search, 16 cases of mediastinal EES had been reported prior to our current case (5–15). Including our case, the total number of reported patients is 17, comprising 10 males (59%) and 7 females (41%), with a mean age of 36 years (range 11–66). Most patients initially presented with chest or back pain, dyspnea, cough, or wheezing. Among the available data, tumors were typically large at diagnosis, with maximum diameters ranging from 3.2 cm to 19.0 cm. On CT, the lesion commonly appears as an irregular soft-tissue mass with areas of low-density cystic necrosis and demonstrates heterogeneous enhancement after contrast. MRI findings have been described in only a few reports, usually showing mildly increased signal intensity on T2-weighted imaging. Most patients received chemotherapy, while a minority underwent surgical resection or received adjuvant therapy after surgery. Overall, the prognosis appears poor, with some patients dying during a short follow-up period.

**Table 2:** Clinical and imaging features of the cases of primary mediastinal Ewing’s sarcoma.

Author, year, country	Gender /age	Main symp toms	Location	CT				MRI		Metastases at diagnosis	Treatment	Recurrence	Follow- up/ (months)	DOI
				MD (cm)	Cystic necrosis	Calcification	CECT	T1WI	T2WI					
Reali A/ 2013/Italy (5)	F/66	chest pain, difficulty breathing	right anterior superior mediastinum	NA	(+)	(-)	mild uneven enhancement	NA	NA	no	ChT+RT	lung, brain	7/live with diease	10.1002/ccr3.4857
Li X/2023 /China (6)	F/15	chest pain, cough and wheezing	left anterior superior mediastinum	15.7	(+)	(-)	mild uneven enhancement	NA	NA	yes/pleura	ChT	NA	3/live	10.1097/MD.0000000000002725
Ata F/2021 /Qatar (7)	M/16	cough and dyspnea	left superior mediastinum	18.0	(+)	(-)	uneven progressive enhancement	NA	NA	yes/pleura	ChT	NA	0.5/die	10.3389/fonc.2023.1074378
Zhang W/ 2010/China (8)	M/47	NA	right anterior superior mediastinum	NA	(+)	(-)	mild uneven enhancement	NA	NA	no	ChT	NA	3/die	10.3389/fonc.2022.1020339
Zhang W/ 2010/China (8)	M/14	NA	right anterior superior mediastinum	13.3	(+)	(-)	mild uneven enhancement	NA	NA	bone	ChT	no	NA/live	10.3389/fonc.2022.1020339
Zhang W/ 2010/China (8)	F/51	NA	anterior superior mediastinum	NA	(+)	(-)	mild uneven enhancement	NA	NA	bone	ChT	no	NA/live	10.3389/fonc.2022.1020339
Zhang W/ 2010/China (8)	M/35	NA	right anterior superior mediastinum	3.2	(+)	(-)	mild uneven enhancement	NA	NA	no	ChT	yes	18/die	10.3389/fonc.2022.1020339
Zhang W/ 2010/China (8)	F/33	NA	right anterior superior mediastinum	NA	(+)	(-)	moderate uneven enhancement	NA	NA	no	ChT	no	NA/live	10.3389/fonc.2022.1020339
Zhang W/ 2010/China (8)	M/59	NA	anterior superior mediastinum	NA	(+)	(-)	mild uneven enhancement	NA	NA	no	ChT	no	NA/live	10.3389/fonc.2022.1020339
Cui M/2022 /China/ (9)	M/66	chest tightness	right anterior mediastinum	NA	(+)	(-)	uneven progressive enhancement	NA	NA	yes/LNs	surgery	yes	1/die	10.1016/j.arbres.2012.02.020
Caltavituro A/2023/Italy (10)	F/31	cough and dyspnea	left anterior mediastinum	19.0	(+)	(-)	uneven enhancement	NA	NA	no	surgery, ChT, stem cell transplantation	no	NA/live	10.1515/biol-2022-0669
Manduch M/2008/Canada (11)	F/24	back pain	left upper posterior mediastinum	5.0	NA	NA	NA	NA	slightly high signal	no	surgery, ChT	no	12/live	10.3892/ol.2014.2788
Bae SH/ 2016/Korea (12)	M/42	chest pain	left mediastinum	15.0	(+)	(-)	uneven enhancement	NA	NA	yes/LNs	surgery	yes/bone	4/live with disease	10.1177/030089160809400623
Liu M/2015 /China (13)	F/51	chest pain	posterior mediastinum	NA	(+)	(+)	uneven enhancement	NA	NA	no	ChT+RT	no	8/live	10.4103/1817-1737.109834
Su C/2023 /China (14)	M/32	chest and back pain	right upper posterior mediastinum	4.0	(+)	(-)	uneven enhancement	NA	slightly high signal	no	surgery	no	12/live	10.3389/fonc.2023.1290603
Halliday J /2010/UK (15)	M/16	dyspnea	upper posterior mediastinuml	7.6	(-)	(-)	NA	NA	NA	NA	NA	NA	NA	10.1016/j.athoracsur.2010.01.083
Our case	M/11	left shoulder pain	posterior mediastinum	9.6	(+)	(-)	uneven progressive enhancement	equal T1 signals	slightly long T2 signals	no	surgery, ChT	no	41/live with disease	NA

M, male; F, female; CT, computed tomography; MRI, magnetic resonance imaging; ChT, chemotherapy; CECT, contrast-enhanced computed tomography; RT, radiotherapy; LNs, lymph nodes; MD, maximum diameter; T1WI, T1 weighted imaging; T2WI, T2 weighted imaging; (+), positive; (-) negative; NA, not applicable.

## Discussion

EES is a small round cell malignant tumor of soft tissue, which accounts for only 1.1% of soft tissue malignant tumors (16). EES is more common in the paravertebral regions, lower extremities, and chest wall, while is rare in the mediastinum. Our case and literature review reveals that mediastinal EES has a wide range of onset ages, ranging from 11 to 66 years, which is slightly different from the literature reports that ES is more common in children and adolescents (3). Patients with tumors in the anterior mediastinum usually present with chest pain, cough, dyspnea, and wheezing, while patients with tumors in the posterior mediastinum often present with back pain. In the patient we reported, the tumor was located in the left posterior superior mediastinum, and the clinical manifestation was left shoulder pain, which may be related to tumor compression or invasion of surrounding tissue.

Imaging examinations including CT and MRI play a crucial role in the diagnosis, staging, and treatment monitoring of EES. Previous studies have revealed that EES is often a large, single, round or irregular solid mass, often accompanied by cystic degeneration and necrosis, but calcification is rare, with an incidence rate of less than 10% (17, 18). On MRI, it mainly showed low to equal signal on T1WI, mixed equal signal or slightly high signal on T2WI, and most of the tumors were cystic solid, in which the cystic part showed low signal on T1WI and high signal on T2WI, and showed obvious uneven enhancement on contrast-enhanced scan (19, 20). The patient we reported presented as a large volume circular mass on CT, with low-density cystic necrotic areas visible inside. On MRI, it showed equal muscle signal on T1WI and uneven slightly high signal on T2WI, which is consistent with the imaging findings of EES reported in the literature. To further understand its characteristics, a systematic review of published literature on EES occurring in the mediastinum were conducted and the results showed similar imaging findings. Moreover, in the cases of arterial phase and venous phase contrast enhancement scan, they all showed progressive enhancement, that is, the lesions were mild to moderate enhancement in the arterial phase, and further enhancement in the venous phase, suggesting that this sign has a certain relative specificity for the diagnosis of mediastinal EES.

The mass in the patient we reported originated from the posterior mediastinum, and the imaging differential diagnosis mainly included neurogenic tumor and germ cell tumor. Neurogenic tumors are the most common tumor in the posterior mediastinum. The typical imaging findings of neurogenic tumors is a round or spindle-shaped soft tissue density mass protrusion to one side of the mediastinum with scattered calcified or necrotic liquefaction areas (21). Benign neurogenic tumors have clear boundaries and uniform enhancement on contrast-enhanced scan, while malignant tumors often have unclear boundaries and obvious uneven enhancement (22), which are different from the imaging findings of EES. Germ cell tumors occurring in the mediastinum also often present as large, uneven soft tissue density masses. In addition to being prone to cystic degeneration and necrosis, they are often accompanied by calcification and bleeding, which are less common in EES (23). Moreover, through our case presentation and literature review, it was found that some EES can secondary involve the bone surface and be accompanied by adjacent cortical erosion. Therefore, it should also be differentiated from bone EWS in the corresponding area, and the difference mainly based on whether the bone marrow cavity is involved (18).

The diagnosis of EES is mainly based on histopathology and immunohistochemistry. Microscopically, it is mainly composed of tightly arranged small round tumor cells in the form of cords, nests or clumps (4). Immunohistochemistry showed that the tumor cells positively expressed CD99 and vimentin, some cases could positively expressed NSE, but negatively expressed S100, neurofilament protein, desmin and keratin (24). Previous study has been reported that NKX2.2 is diffusely positive in EES tissues, significantly higher than in other small round cell tumors, so can be used as a new marker for the diagnosis and differential diagnosis of EES (25). Moreover, CD99 positive expression also helps differentiate EES from other small round cell tumors (25, 26). The histopathological examination of this patient showed that the tumor was composed of small round cells of similar size and shape under the microscope, and immunohistochemical results showed that the tumor cells expressed CD99, NKX2.2 and vimentin positively, which was consistent with the diagnosis of EES.

As EWS is a highly invasive tumor, the National Comprehensive Cancer Network guidelines recommend that any EWS should be treated in combination with local therapy (surgery and/or radiotherapy) and chemotherapy (27). Surgical resection is considered a critical therapeutic intervention when feasible, as complete resection of the tumor may alleviate symptoms and enhance the effectiveness of subsequent adjuvant therapies (9). Studies have revealed that preoperative chemotherapy combined with surgical resection of the tumor can significantly improve the prognosis of patients, and the proven effective chemotherapy drugs for EWS include vincristine, doxorubicin, cyclophosphamide, ifosfamide and etoposide (11, 28). For recurrent or refractory cases, second-line regimens such as temozolomide/irinotecan and cyclophosphamide/topotecan are widely used (29). Temozolomide inhibits tumor proliferation, induces tumor cell apoptosis, and suppresses angiogenesis by interfering with DNA synthesis and repair processes, while irinotecan further enhances cytotoxicity in this combination (30). Cyclophosphamide, as an alkylating agent, interferes with DNA replication, inhibits tumor cell growth and reproduction; Topotekang supplements its effects and jointly improves anti-tumor efficacy (31). A published randomized clinical trial revealed that chemotherapy with VDC (vincristine, doxorubicin, cyclophosphamide) plus IE (isocyclophosphamide plus etoposide) was more effective, less toxic, and more convenient than VIDE for all stages of newly diagnosed EWS and should be the standard of first-line care for all patients with EWS (32). Compared to primary bone EWS, EES has a better prognosis, while about 25% of patients with the disease have already developed metastases at the time of diagnosis, so its 5-year survival rate is still below 30% (27). According to the literature review, the prognosis of EES in mediastinum is still poor. Most patients did not have the opportunity of surgical resection due to excessive volume or distant metastasis at the time of diagnosis, resulting in the death of some patients in a short period (0.5–18 months). In addition, studies have reported that the prognosis of EWS is related to the age of the patient at the time of diagnosis. The older the patient, the higher the probability of recurrence, while patients under the age of 16 have a relatively good prognosis (33). The current patient reported by us experienced multiple recurrences or metastases during the follow-up period, further confirming the high malignancy and poor prognosis of this tumor.

The current study has several limitations. First, our findings are based on a case from a single institution, and evidence regarding second-line treatment for metastatic or recurrent EWS remains limited. Given the rarity of this disease, future research should validate these findings through multicenter studies with larger sample sizes. Second, amrubicin was administered orally despite the recommended intravenous route, due to the patient’s poor compliance, which may have compromised its therapeutic efficacy. Third, since the expected effect of the first-line treatment regimen used by this patient at the initial diagnosis was not very significant, when the disease recurred, the patient received a second-line treatment regimen at another hospital. Furthermore, part of the treatment was delivered at an external hospital, leading to incomplete documentation of certain clinical details. These factors collectively represent limitations that should be considered when interpreting the results. However, through our case report and a detailed review of relevant literature, our study comprehensively summarized the imaging and clinical features as well as the treatment experience of this rare disease, mediastinal EES, providing a reference for clinicians in their future work.

## Conclusion

In conclusion, this article presents a rare case of EWS arising at an unusual location-the mediastinum. Due to the rarity of this disease and the non-specific nature of its clinical manifestations, it is difficult to obtain an accurate diagnosis before surgery. Our study suggests that when imaging findings presents as large, round or lobulated masses with soft-tissue density in the mediastinum, frequently accompanied by low-density cystic necrotic areas, and MRI demonstrates T1-weighted and T2-weighted signal intensities that are isointense or slightly hyperintense relative to muscle, along with progressive enhancement on contrast-enhanced scans, EWS should be considered as a potential diagnosis.

## Data Availability

The datasets presented in this article are not readily available because of ethical and privacy restrictions. Requests to access the datasets should be directed to the corresponding author/s.
